# 4340 Piloting Implementation and Dissemination of Best Practice Guidelines Using BPM+Health

**DOI:** 10.1017/cts.2020.418

**Published:** 2020-07-29

**Authors:** James McClay, Pawan Goyal

**Affiliations:** 1 University of Nebraska Medical Center - Great Plains IDeA-CTR; 2 American College of Emergency Physicians

## Abstract

OBJECTIVES/GOALS: Clinical translational studies inform clinical practice patterns through dissemination of clinical practice guidelines (CPG). In EM practices change to rapidly for timely local EHR implementation. We test the OMG BPM+Health specification for rapid deployment of best practices relevant to EM. METHODS/STUDY POPULATION: The OMG Business Process Management for Healthcare (BPM+Health) specification combines BPMN™ with Case Management Model and Notation (CMMN™) and Decision Model and Notations (DMN™) to “disseminate and leverage evidence-based best-practices at the point of care.” The American College of Emergency Physicians (ACEP) Board-certified Emergency Physicians modeled practice guidelines in the BPM+ modeling language during on-line meetings. Two common emergency conditions were selected for initial pilot testing: 1) evaluation and treatment of first trimester bleeding in pregnant patients, and 2) the evaluation and treatment of non-traumatic low back pain. RESULTS/ANTICIPATED RESULTS: The protocols were successfully modeled during four on-line meetings in less than 2 months. Process steps from initial evaluation to disposition were implemented using BPMN™. When clinicians need to evaluate the patient to collect data for decision making the inputs and outputs were modeled in CMMN™. Decision logic is represented as DMN™. The software tool linked the components for easy browsing and authoring the logic. The Physicians easily followed the displayed logic. The practice recommendations from each policy were successfully modeled, using the standard BPM+ notation to support rapid implementation in EHRs. Detailed implementation specifications will be shared. DISCUSSION/SIGNIFICANCE OF IMPACT: This pilot project demonstrated the feasibility of the OMG approach to solving Clinical Practice Guideline Implementation and Dissemination Barriers. Ongoing work by involved specialty societies will be necessary to demonstrate the scalability and sustainability of this approach.

